# Integrating Omic Technologies into Aquatic Ecological Risk Assessment and Environmental Monitoring: Hurdles, Achievements, and Future Outlook

**DOI:** 10.1289/ehp.0900985

**Published:** 2009-08-17

**Authors:** Graham Van Aggelen, Gerald T. Ankley, William S. Baldwin, Daniel W. Bearden, William H. Benson, J. Kevin Chipman, Tim W. Collette, John A. Craft, Nancy D. Denslow, Michael R. Embry, Francesco Falciani, Stephen G. George, Caren C. Helbing, Paul F. Hoekstra, Taisen Iguchi, Yoshi Kagami, Ioanna Katsiadaki, Peter Kille, Li Liu, Peter G. Lord, Terry McIntyre, Anne O’Neill, Heather Osachoff, Ed J. Perkins, Eduarda M. Santos, Rachel C. Skirrow, Jason R. Snape, Charles R. Tyler, Don Versteeg, Mark R. Viant, David C. Volz, Tim D. Williams, Lorraine Yu

**Affiliations:** 1 Environment Canada, Pacific Environmental Science Centre, Vancouver, British Columbia, Canada;; 2 U.S. Environmental Protection Agency, Duluth, Minnesota, USA;; 3 Clemson University, Clemson, South Carolina, USA;; 4 National Institute of Standards and Technology, Hollings Marine Laboratory, Charleston, South Carolina, USA;; 5 U.S. Environmental Protection Agency, Gulf Ecology Division, Gulf Breeze, Florida, USA;; 6 The University of Birmingham, Birmingham, West Midlands, United Kingdom;; 7 U.S. Environmental Protection Agency, Athens, Georgia, USA;; 8 Glasgow Caledonian University, Glasgow, Scotland, United Kingdom;; 9 University of Florida, Gainesville, Florida, USA;; 10 Health and Environmental Sciences Institute, Washington, DC, USA;; 11 Stirling University, Stirling, Scotland, United Kingdom;; 12 University of Victoria, Greater Victoria, British Columbia, Canada;; 13 Syngenta Crop Protection Canada Inc., Guelph, Ontario, Canada;; 14 Okazaki National Research Institutes, Okazaki, Aichi, Japan;; 15 Ecogenomics Inc, Kurume, Fukuoka, Japan;; 16 Centre for Environment, Fisheries and Aquaculture Science, Weymouth, Dorset, United Kingdom;; 17 Cardiff University, Cardiff, Wales, United Kingdom;; 18 Johnson & Johnson, New Brunswick, New Jersey, USA;; 19 Environment Agency, Bristol, United Kingdom;; 20 U.S. Army Corps of Engineers, Vicksburg, Mississippi, USA;; 21 Exeter University, Exeter, Devon, United Kingdom;; 22 Astrazeneca, Brixham Environmental Laboratory, Devon, United Kingdom;; 23 Procter and Gamble, Cincinnati, Ohio, USA;; 24 Syngenta Crop Protection Inc., Greensboro, North Carolina, USA

**Keywords:** environment, environmental monitoring, fish, metabolomics, microarray, regulatory toxicology, transcriptomics

## Abstract

**Background:**

In this commentary we present the findings from an international consortium on fish toxicogenomics sponsored by the U.K. Natural Environment Research Council (Fish Toxicogenomics—Moving into Regulation and Monitoring, held 21–23 April 2008 at the Pacific Environmental Science Centre, Vancouver, BC, Canada).

**Objectives:**

The consortium from government agencies, academia, and industry addressed three topics: progress in ecotoxicogenomics, regulatory perspectives on roadblocks for practical implementation of toxicogenomics into risk assessment, and dealing with variability in data sets.

**Discussion:**

Participants noted that examples of successful application of omic technologies have been identified, but critical studies are needed to relate molecular changes to ecological adverse outcome. Participants made recommendations for the management of technical and biological variation. They also stressed the need for enhanced interdisciplinary training and communication as well as considerable investment into the generation and curation of appropriate reference omic data.

**Conclusions:**

The participants concluded that, although there are hurdles to pass on the road to regulatory acceptance, omics technologies are already useful for elucidating modes of action of toxicants and can contribute to the risk assessment process as part of a weight-of-evidence approach.

The goal of this consortium (Fish Toxicogenomics—Moving into Regulation and Monitoring, held 21–23 April 2008 at the Pacific Environmental Science Centre, Vancouver, BC, Canada) was to assess current developments leading to the incorporation of omic technologies into environmental risk assessment and environmental monitoring, particularly in relation to aquatic ecotoxicogenomics. Participants recognized that omic tools and associated end points are already significantly improving our understanding of how individual chemicals and mixtures affect organisms and could ultimately influence risk assessment and environmental management. Although a significant amount of basic research and validation is needed before omic end points are incorporated as complementary data for routine assessments of environmental risk, participants generally agreed that there are no roadblocks for omics technology per se, but there are hurdles along the road of discovery, acceptance, and implementation of omic end points. Given the context of the workshop, it is important to note that “the successful incorporation of toxicogenomics into regulatory frameworks may someday be regarded as the most important intellectual and practical contribution from this generation of ecotoxicologists” (Ankley et al. 2006).

## Benefits and Successful Applications of Omics in Ecotoxicology and Ecological Risk Assessments

### Historical challenges and recent developments for regulatory implementation

Previous publications and workshops (e.g., Ankley et al. 2006; Boverhof and Zacharewski 2006) have discussed the potential application of omic technologies to risk assessment. The use of omic technology in toxicology (toxicogenomics) was initiated after the development of the first high-density techniques (microarrays). However, excitement surrounding this new technology generated “hype” that yielded unrealistic expectations of the timeline for incorporation into risk assessment. There is now a more realistic understanding of the potential contribution of omics to toxicology [National Research Council (NRC) 2007]. A multilevel systems biology approach to safety assessment—combining molecular- (including mRNA, protein, and metabolites), cellular-, tissue-, individual-, and population-level data—represents a powerful new multidisciplinary approach that identifies biomarkers with much-improved predictive capacity.

Many initial concerns and difficulties have been overcome. The high cost of microarrays imposed severe restrictions on the number of doses, replicates, and time points assessed after chemical administration to biological systems. As a consequence, reported omic responses frequently reflected pathological change with no evident predictive value. Methodology has now been improved and costs reduced, and microarrays are commercially available for a range of species. In the context of fish transcriptomics, a special issue of the *Journal of Fish Biology* (Miller and Maclean 2008) reported the progress that has been made with nonmodel organisms.

### Successful prospective and diagnostic case studies

Transcriptomic experiments in aquatic toxicology have been diverse—encompassing different microarray platforms, test species, and exposure routes—emphasizing their use as case studies rather than standardized tools. This wide-ranging approach contributes to the elucidation of mechanisms of toxicity, including dose–response relationships, differential species sensitivity, and classification of chemical- specific biological responses. This approach also provides leads for identification of novel biomarkers of exposure and adverse effect.

Omic and bioinformatic tools offer substantial promise for discovery of gene, protein, and/or metabolite alterations indicative of the mode of action (MOA) of chemicals and improved understanding of mechanisms in prospective studies (Ankley et al. 2006). Knowing the MOA can reduce uncertainties in chemical risk assessments, providing, for example, a basis for extrapolating effects across species (Benson and Di Giulio 2007). There is ongoing debate as to the appropriate role of biomarker data in ecological risk assessments (Forbes et al. 2006). Historically, most biomarker data employed in ecotoxicology indicated exposure but had limited ability to predict deleterious effects meaningful to risk assessment, namely, survival, growth and development, and reproduction (Forbes et al. 2006), largely because of a lack of mechanistic knowledge concerning linkages between molecular alterations and outcomes in the whole organism. Ideally, omics data would reflect both the MOA and deleterious outcome(s). To achieve this, the cascade of pathways associated with toxicity must be defined, from a molecular initiating event (e.g., receptor binding) through subsequent biological alterations (reflected by omic and cellular changes) that culminate in a deleterious outcome (NRC 2007). However, there is also potential for a contribution to understanding ecological impacts. Furthermore, omic approaches can contribute to the reduction of animal use and the severity of treatments, as more subtle changes can be identified and a more complete assessment of the health of individual animals or cell cultures can be achieved.

A recent example of how a toxicity pathway approach can be used to establish quantitative linkages across biological levels of organization was provided by Miller et al. (2007) and Ankley et al. (2008a), who investigated the consequences of molecular changes in the fish hypothalamic–pituitary–gonadal (HPG) axis in terms of reproductive and population effects. Production of vitellogenin (VTG), an oocyte lipoprotein produced in the liver of oviparous female vertebrates, can be affected by a range of signaling events that alter steroid hormone production and activity. Analysis of an integrated data set derived from fathead minnow (*Pimephales promelas*) reproduction studies, with five chemicals that decrease VTG and fecundity but affect the HPG axis through different discrete mechanisms, demonstrated robust associations between steroid and VTG concentrations in female fish. This was predictive of egg production and, via modeling, could be used to tentatively forecast fathead minnow population status. Thus, through understanding the biological pathways leading to vitellogenesis, mechanistic molecular responses were successfully related to potential adverse outcomes meaningful to risk assessors.

Omics data can be used in diagnostic studies to determine the efficacy of pollution remediation as part of a weight-of-evidence approach (Roling et al. 2007). Furthermore, omic profiling can be used to identify chemical causation of effects induced by complex mixtures (Garcia-Reyero et al. 2008a). For example, in studies with the fathead minnow, Filby et al. (2007) applied multiple quantitative polymerase chain reaction (PCR) to identify diagnostic signatures from different chemicals that induce the same phenotypic effects. The authors identified common features in the responses of fish exposed to estrogenic wastewater effluent and individual steroid estrogens. These data demonstrate that patterns of gene expression induced by estrogenic effluents, although complex, can be diagnostic for some of the estrogens they contain and could be used by regulators to determine the primary contaminant.

### The utility of omics in studies on environmental sites

The influence of the local environment on the transcriptome or metabolome of an organism can be exploited in environmental monitoring to characterize the effects of anthropogenic stressors such as pollution. In European flounder (*Platichthys flesus*) (Falciani et al. 2008), transcriptomics and genetic algorithm bioinformatic approaches were used to predict the site of origin of fish from the environment based on stress-responsive genes. Thus, although gene expression is affected by many environmental factors, a subset of genes with altered expression can inform on stress responses. The potential utility here is to improve biomarker identification and to identify patterns of gene expression associated with different types of pollution. Bundy et al. (2007) sampled an earthworm species (*Lumbricus rubellus*) from seven sites with different levels of metal contamination. Using nuclear magnetic resonance (NMR) metabolomics, the authors showed that metabolic profiles of the earthworms could resolve individual sites. Despite the confounding influences of site parameters, specific metabolites were correlated with zinc, the major contaminant, across all seven sites. Another NMR metabolomics study involving flounder sampled from industrialized and reference sites in the United Kingdom showed that water composition had a significant effect on the fish liver metabolome (Parsons et al. 2007). Uses of omic data for prognostic and diagnostic studies are summarized in Supplemental Material, Table 1, available online (doi:10.1289/ehp.0900985.S1 via http://dx.doi.org/). These important observations lend support to the implementation of omics as diagnostic tools in ecotoxicology.

## Current Hurdles from a Regulatory Viewpoint

### General technical hurdles in the application of toxicogenomics

Ankley et al. (2006) proposed a time frame for realizing the utility of omics in tiered testing. However, a number of factors limit widespread acceptance for regulatory applications. In addition to complex relationships between omic responses and ecological outcome, standardized, validated exposure assay and analysis procedures are lacking (Ankley et al. 2006). Omics technologies can be viewed as complementary testing procedures that can improve understanding of biological systems and can lead to development of simpler individual assays with defined end points. This approach, although valuable, does not exploit the full capabilities of omics, particularly their open nature, allowing discovery of unexpected changes. Intermediate technologies such as PCR arrays have been proposed for use in clinical diagnosis (Bustin and Mueller 2005) but are not open systems. The validation required for uptake of multibiomarker techniques for routine testing is time consuming and complex. In the clinical setting, some targeted microarray applications have now been approved by the U.S. Food and Drug Administration and the European Union for diagnostic use. These include the Amplichip CYP450 (Roche, Indianapolis, IN, USA) for genotyping of human cytochromes P450 and Mammaprint (Agendia, Amsterdam, the Netherlands), a gene-expression microarray for breast cancer prognosis (Glas et al. 2006). Mammaprint validation required the testing of over 1,000 patient samples in 12,000 assays, highlighting the effort and investment necessary for such accreditation. Validation of an assay is a complex procedure encompassing the determination of its reliability and relevance. Hartung et al (2004) discussed a modular approach to validating alternative tests as part of an initiative by the European Centre for Validation of Alternative Methods. This approach [see Supplemental Material, Figure 1 (doi:10.1289/ehp.0900985.S1)] is applicable in general terms to omics-based assays. Key to this procedure is defining a relevant end point. Biomarkers can be of exposure or of effect, and the choice between these biomarkers and the end points they aim to predict must be informed by the requirements of the regulators.

Corvi et al. (2006) suggested requirements for validation of transcriptomics in regulatory toxicology, but it is unlikely that this validation process will be rapid. However, a realistic application of omics techniques may be their use in prescreening chemicals and mixtures for prioritization in further tests (Ankley et al. 2006). The ToxCast program (Dix et al. 2007) employed a diverse selection of tests, including toxicogenomics, and showed the potential application of this approach. The interpretation of omic data is highly reliant on advanced computational and statistical methods that are still being developed. Although quality assurance procedures are paramount, more flexibility is permitted at the prescreening tier. The U.S. Environmental Protection Agency (EPA 2002) currently accepts toxicogenomics data as part of a weight-of-evidence approach for establishing mechanisms of toxicity for regulated substances. Data capture and archiving are essential mechanisms for highlighting and avoiding the pitfalls of inappropriate experimental design, such as the introduction of systematic variation during omics experiments. Although transcriptomics databases are well established, toxicology-specific omics databases are now emerging (Waters et al. 2008).

Environmental metabolomics recently benefited from the first interlaboratory intercomparison exercise to evaluate the accuracy, precision, and efficacy of ^1^H NMR metabolomics (Viant et al. 2009). Flounder liver extracts from contaminated and reference sites were analyzed, and multivariate statistical analyses confirmed high reproducibility across all seven laboratories involved in the study. Furthermore, the same metabolic biomarkers used to differentiate fish from the two sites were discovered by all the laboratories [see Supplemental Material (doi:10.1289/ehp.0900985.S1)]. For transcriptomics in fish, diversity of microarray platforms has precluded interlaboratory comparisons, but interlaboratory microarray comparison has been successful for mammalian species (e.g., Mattes 2008; Shi et al. 2006). It is likely that, in the future, improvements in the technologies for assaying gene expression, such as high-throughput pyrosequencing and digital transcriptomics (Nielsen et al. 2006), will replace microarrays. Already, pyrosequencing allows for fast construction of high-quality oligonucleotide microarrays for nonmodel species (Garcia-Reyero et al. 2008b). The technology is constantly evolving, but there is one key question that must be addressed, whatever technology is in use: How do gene and protein expression and metabolite concentrations relate to ecological outcome? Progress has been made on this question, and initial studies on population bases are now being published [see Supplemental Material, Table 1 (doi:10.1289/ehp.0900985.S1)]. Although biomarkers are valuable in regulatory and monitoring contexts, the meanings of such changes must be clarified to allow efficient use in regulatory decision making (Adelman 2005; Boverhof and Zacharewski 2006).

### Identifying sources of variation and minimizing their effects

Variability in omics data is an ongoing concern, particularly in relation to multiple individual manipulations between biological sampling and data interpretation. Workshop participants identified sources of technical and biological variation and recommended how these should be managed in terms of experimental design [see Supplemental Material (doi:10.1289/ehp.0900985.S1)]. Study design, inadequate sample numbers, and methods of sample acquisition, preparation, storage, processing, and analysis are key areas of possible technical artifacts. Major sources of variability include methods of normalization and statistical interpretation. Careful study design is essential to minimize biological variability intraclass (e.g., stage of reproduction in a control group), thus maximizing interclass differences (e.g., control vs. exposed groups). Interindividual variability within a population is essential for ecological health, and therefore an impact on such variability from a stressor can be very important.

### Training and communication

To advance the application of omics technology into regulatory ecotoxicology and water quality policy, effective scientific communication will be necessary among academia, industry, and regulators (Blunt et al. 2007). The benefits and limitations of omics techniques need to be candidly discussed so that tools with the potentially greatest return on investment (both financial and knowledge based) may be prioritized for utilization. Multidisciplinary workshops allow continued dialogue to inform all stakeholders of developments. These cross-functional meetings also provide researchers with an understanding of the priorities of regulatory authorities to discover practical ways of solving issues.

Advances in omics have significant implications for risk assessment practice and regulatory decision making. The use of genomics technologies generates a large volume of data, and the field of bioinformatics is evolving rapidly to meet data analysis needs. A genomics white paper (U.S. EPA 2004) identified areas likely to be influenced by omics. In that report, the Genomics Task Force recommended that the agency develop training materials and modules to prepare risk assessors and decision makers who will be faced with the challenge of interpreting and applying omics information. Participants in the workshop described here also believed that training was critical for furthering the application of genomics technologies into monitoring and regulation, particularly as a means of interpreting and applying genomics data for risk assessment (Dearfield et al. 2008). Risk assessors must be able to communicate to managers and stakeholders both the underlying science and the interpretive tools and models used to develop the risk assessment. Likewise, it will be important to provide training to risk managers regarding the benefits and limitations of genomics in risk assessments (Haymes et al. 2009).

There is also a need to build capacity within academia, the private sector, and government agencies to implement omic tools and to evaluate omics data, particularly with respect to biological and ecological significance. These institutions will require resources, support, and targeted training to bring scientists and decision makers within their organizations to a point where these tools can be used effectively in regulatory decision making, especially in risk assessment (U.S. EPA 2004). National and subnational programs and agencies should apply strategic hiring practices to recruit individuals who possess omics skills. It would be useful to develop and initiate training in the near future to enable risk assessors and risk managers to evaluate and incorporate omic data into environmental decision making. Initial training could address basic omics concepts, technologies, and potential applications and include the basic steps necessary to interpret and apply genomics data to risk assessment.

### Research needs for regulatory implementation

Research needs were reviewed in two successive Society of Environmental Toxicology and Chemistry Pellston workshops held in 2004 and 2005 (Ankley et al. 2006, 2008b; Benson and Di Giulio 2007). These efforts identified both short- and long-term needs that have not yet been fully addressed because of resource constraints.

The short-term needs identified were *a*) formal standardization and validation of data collection, analysis, and presentation for standard test species; and *b*) generation of libraries of gene expression, proteomic, or metabolite profiling data based on a set of reference chemicals with well-defined, relevant MOAs. As explained above, there have been important advances in recent years in the context of both of these needs. The long-term needs identified were *a*) generation of genome sequence data for ecologically relevant species; and *b*) linkage of molecular and biochemical responses to adverse alterations in survival, growth and development, and reproduction.

Significant advances have also been made toward obtaining data for the development of reference gene expression profiling databases from species commonly used for regulatory assessments (Ankley et al. 2008b), although much work remains. Because toxicogenomics data will be most valuable for predictive toxicology and elucidating toxicologically relevant MOAs for additional chemicals, a future database should be based on toxicity testing and monitoring protocols commonly used for regulatory purposes (e.g., global pesticide registrations), as well as chemicals with well-known MOAs such as 17β-estradiol and dioxin.

### Funding

Chemical production is highest in member countries of the Organisation for Economic Co-operation and Development (OECD), particularly in specialty chemicals and the life science sectors. Moreover, innovation in new chemical development and manufacturing practices is extremely high due to advances in combinatorial chemistry, nanotechnology, and biotechnology. These changes question the sustainability of current approaches to prioritization, monitoring, and risk assessment. It may be difficult to allocate additional resources required to efficiently incorporate and understand omics data, but it is important for programs and agencies to focus on these needs and to ensure that adequate funds and people are brought to bear on this need.

The OECD Environment, Health and Safety Program has started cooperative work for the use of genomic information for risk assessment of chemicals. The scope of this activity is to explore and evaluate regulatory application of genomic methods in chemical hazard/risk assessment. To reduce redundancy and minimize the funding to develop these omic technologies, international cooperation and a common database are essential. Target and cross-species omic information and technologies should be developed to monitor animal species relevant to disparate countries. There are many international examples of initiatives to enhance ecotoxicogenomics, for example, the Ministry of Environment of Japan, the Canadian government’s Interdepartmental Genomics initiative, the U.S. EPA initiatives, and the U.K. Natural Environment Research Council Postgenomics and Proteomics research program.

A considerable investment in the generation and curation of appropriate reference exposure data is required to address the toxicogenomics needs. For example, for most chemical registrations, chronic aquatic toxicity data are required for one freshwater fish species (early life-stage toxicity), one freshwater invertebrate species (full life-cycle toxicity), and, in most cases, one saltwater fish species (early life-stage toxicity) and one saltwater invertebrate species (full life-cycle toxicity). This leads to a large number of Good Laboratory Practice–compliant studies, and such a reference database would likely require ~ $10 million over 3–5 years. Although this is a substantial cost, a reference database is arguably the only means for successful and appropriate implementation of toxicogenomics data into the current ecological risk assessment paradigm. Without a database to compare chemicals with unknown MOAs, the risk assessor will not be able to interpret the significance of the gene expression responses within the context of characterizing ecological risk, because many gene expression changes are not anchored to adverse effect and risk assessment requires knowledge of the MOA and dose–response relationship. A good example of the utility of a comprehensive database is ToxRefDB, the Toxicity Reference Database (Martin et al. 2009), which contains mammalian toxicity data.

The most feasible way forward would be funding through a multistakeholder consortium, such as that achieved in the ACToR (Aggregated Computational Toxicology Resource) database (Judson et al. 2008). The final reference database would be open-source and accessible through a web site. The reference database could also be integrated into a tool similar to the U.S. Food and Drug Administration’s ArrayTrack to allow regulatory agencies to easily manage, analyze, and interpret omics data submitted by registrants or other government or academic laboratories using similar ecological species, testing protocols, and microarray, proteomic, or metabolomic platforms.

## Conclusions

Omic technologies have advanced over recent years and continue to become more efficient, data-rich, and economical in use. Proof of principle has been achieved in terms of potential application to environmental toxicology, specifically the assessment of environmental pollution impacts in nonmodel organisms. Increasing the use of omics technology in chemical risk assessment and environmental monitoring requires an expanded ecotoxicogenomics reference database and a better understanding of the relationships between specific responses and biomarkers to ecological adverse events. Through improved communication between the sectors, the aim of assisting in regulatory decisions can be expedited through the use of omic techniques.
